# Serum Endocan as a Novel Biomarker of Cerebral Ischemia–Reperfusion Injury in a Rat Model

**DOI:** 10.3390/biomedicines14061240

**Published:** 2026-05-29

**Authors:** Mehmet Özgür Özates, Kadir Çetinkaya, Yasar Ünsal, Hümeyra Kullukçu, Oktay Gürcan, Atilla Kazancı, Evrim Önder, Tuba Saadet Deveci Bulut, Ahmet Gürhan Gürcay

**Affiliations:** 1 Neurosurgery Department, Yıldırım Beyazıt University, 06000 Ankara, Türkiye; dr.mehmetozgurozates@gmail.com (M.Ö.Ö.); oktaygurcan@gmail.com (O.G.); atillakazanci@gmail.com (A.K.); drgurcay@gmail.com (A.G.G.); 2 Neurosurgery Department, Hitit University Corum Erol Olcok Training and Research Hospital, 19000 Corum, Türkiye; 3 Neurosurgery Department, Bilkent City Hospital, 06000 Ankara, Türkiye; yaasar40@gmail.com; 4 Neurosurgery Department, Mersin Silifke State Hospıtal, 33010 Mersin, Türkiye; humeyrakullukcu@hotmail.com; 5 Pathology Department, Ankara Etlik City Hospital, 06000 Ankara, Türkiye; evrimin@yahoo.com; 6 Department of Biochemistry, Gazi University Faculty of Medicine, 06000 Ankara, Türkiye; tsdbulut@gmail.com

**Keywords:** endocan, ischemia–reperfusion injury, biomarkers, endothelial dysfunction, rodent model

## Abstract

**Background:** Cerebral ischemia–reperfusion (I/R) injury is a significant contributor to mortality and long-term disability following ischemic stroke. Despite advances in neuroimaging, there remains a critical need for non-invasive, sensitive circulating biomarkers for early diagnosis and management. Endocan, a soluble proteoglycan secreted by activated endothelial cells, has been implicated in various vascular inflammatory conditions, but its specific role as a biomarker for cerebral I/R injury in rodent models requires further elucidation. **Methods:** Sixteen adult male Sprague Dawley rats were randomly assigned to either a sham (*n* = 8) or an ischemia–reperfusion (I/R) group (*n* = 8). Cerebral I/R injury was induced by temporary bilateral common carotid artery occlusion for 10 min, followed by reperfusion. Serum endocan levels were quantified using ELISA at baseline (0 min) and 6, 24, and 48 h post-reperfusion. Histopathological evaluation of hippocampal neuronal degeneration was performed at 48 h using a four-point grading system by blinded neuropathologists. Statistical analyses included independent samples *t*-tests, one-way repeated measures ANOVA, and Spearman’s rank correlation. **Results:** Baseline serum endocan levels did not differ between groups (*p* = 0.814). However, in the I/R group, endocan concentrations were significantly elevated compared to the sham group at 6 h (*p* < 0.005), 24 h (*p* < 0.001), and 48 h (*p* < 0.001). Intra-group analysis of the I/R cohort revealed a significant rapid elevation in endocan levels relative to baseline at 6 h (*p* = 0.003), followed by a gradual decline at 24 h (*p* < 0.001) and 48 h (*p* = 0.028), remaining significantly elevated above baseline at all time points. Histopathological examination showed significantly greater neuronal degeneration in the I/R group (median score = 2.5) compared to the sham group (median score = 0; *p* < 0.001). A strong positive correlation was observed between serum endocan levels at 48 h and hippocampal neuronal degeneration scores within the I/R group (Spearman’s *ρ* = 0.857, [95% CI: 0.482–0.968]; *p* = 0.007). **Conclusions:** Serum endocan demonstrates high levels following cerebral I/R injury in a rodent model, correlating strongly with the severity of hippocampal neuronal damage. These findings suggest that serum endocan is a sensitive and biologically relevant circulating biomarker for cerebral I/R injury, holding potential for non-invasive monitoring of endothelial dysfunction and secondary injury processes in acute ischemic stroke.

## 1. Introduction

Cerebral stroke remains one of the leading causes of mortality and long-term disability worldwide, with ischemic stroke accounting for approximately 70% to 90% of all cases [1]. The central nervous system is exceptionally sensitive to hypoxia, and brain ischemia triggers a cascade of biochemical changes, including bioenergetic failure, ion homeostasis disruption, and ischemic acidosis [2]. The primary clinical objective in managing acute ischemic stroke is the rapid restoration of blood flow through intravenous thrombolysis or mechanical thrombectomy [3]. However, the process of reperfusion itself can paradoxically exacerbate tissue damage, a phenomenon known as cerebral ischemia–reperfusion (I/R) injury [4]. This complex pathological process involves oxidative stress, neuroinflammation, calcium overload, and the disruption of the blood–brain barrier (BBB), leading to severe neuronal death and neurological dysfunction [5].

Despite the high sensitivity and specificity of neuroimaging techniques such as Computed Tomography (CT) and Magnetic Resonance Imaging (MRI) in diagnosing brain damage, these methods are often costly, require specialized infrastructure, and may fail to identify early-stage pathologies [6]. Consequently, there is an urgent need for non-invasive, cost-effective, and sensitive circulatory biomarkers to aid in the early diagnosis and management of acute cerebrovascular events. While several proteins such as neuron-specific enolase (NSE), S100B, and glial fibrillary acidic protein (GFAP) have been investigated as markers of neuronal and glial damage, the identification of biomarkers reflecting endothelial activation and dysfunction in the context of I/R injury remains a critical area of exploration [7].

Endocan, also known as endothelial cell-specific molecule-1 (ESM-1), is a soluble dermatan sulfate proteoglycan primarily secreted by activated vascular endothelial cells. Its expression is significantly upregulated by pro-inflammatory cytokines and angiogenic factors, and it plays a pivotal role in the inflammatory response by modulating leukocyte migration and cell adhesion molecules [8,9]. Elevated serum levels of endocan have been associated with various vascular and inflammatory conditions, including hypertension, atherosclerosis, and coronary artery disease [10].

Recent studies have proposed endocan as a potential marker for silent brain infarction and a predictor of short-term adverse outcomes in patients with large-artery atherosclerotic stroke [11]. Furthermore, experimental models of spinal cord injury have demonstrated that serum endocan levels correlate with histopathological severity and secondary injury mechanisms such as inflammation and ischemia [12]. However, the specific temporal expression of serum endocan and its role as a novel biomarker for cerebral ischemia–reperfusion injury in rodent models require further elucidation, as existing human studies on ischemic cerebrovascular disease have yielded mixed results regarding its prognostic value [13].

The aim of this study is to investigate serum endocan levels as a novel circulating biomarker of cerebral I/R injury in a rodent model. As an exploratory pilot study, this research is designed to provide preliminary evidence regarding the temporal profile and biological relevance of endocan in the context of I/R-induced brain injury. By evaluating the relationship between endocan levels and the severity of I/R-induced brain damage, we seek to generate hypothesis-driven insights into the potential of endocan as a diagnostic and prognostic tool for monitoring endothelial dysfunction and secondary injury in acute ischemic stroke.

## 2. Materials and Methods

### 2.1. Ethics Statement

All animal-related experimental procedures were performed at the Kobay Experimental Animal Laboratory in compliance with established national regulations and internationally recognized standards for animal welfare and ethical conduct. The study protocol received formal approval from the Kobay Experimental Animals Local Ethics Committee (approval No: 405, 8 July 2019). Throughout the study, all possible measures were taken to reduce the number of animals utilized and to alleviate potential distress. Animal handling, housing conditions, and all experimental interventions were carried out in accordance with the ARRIVE (Animal Research: Reporting of In Vivo Experiments) guidelines, ensuring rigor, reproducibility, and transparency in the reporting of in vivo research.

### 2.2. Experimental Animals and Study Design

Sixteen adult male Sprague Dawley rats, weighing 200–250 g, were ultimately included in this study. Although twenty animals were initially allocated, four were excluded prior to randomization according to predefined criteria. Specifically, two animals were excluded due to unsuccessful induction of cerebral ischemia. The success of the model was verified by the presence of neurological deficits (assessed via a 5-point neurological deficit scale adapted from Longa et al. [14], where a score ≥ 1 was required for inclusion) and subsequent histopathological confirmation of neuronal degeneration in the hippocampal CA1 region. Animals in the I/R group that failed to exhibit these characteristic ischemic changes upon post-mortem evaluation were excluded from the final analysis. In addition, two animals were excluded because of clinical evidence of infection during the experimental period, characterized by reduced activity, piloerection, and body weight loss exceeding 10% of baseline. All exclusion procedures were conducted by an investigator blinded to group allocation.

**Figure 1:**
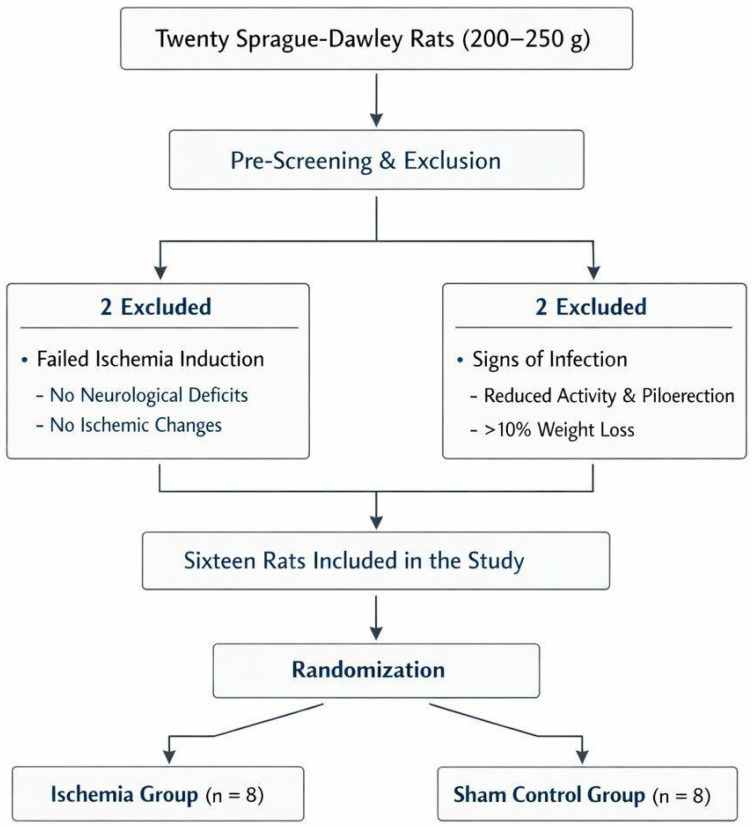
Experimental animal flow diagram.

Following these exclusions, the remaining sixteen animals were randomly assigned to two experimental groups (*n* = 8 per group) for subsequent analyses (Figure 1).

Group 1: Sham (control) group (*n* = 8)

Animals underwent identical anesthesia and surgical preparation without induction of ischemia.

Group 2: Ischemia–reperfusion (I/R) group (*n* = 8)

Transient cerebral ischemia was induced using a standardized experimental protocol, followed by reperfusion.

Given the exploratory nature of this study, no formal a priori sample size calculation was performed. However, post hoc power analyses were conducted to evaluate the robustness of the principal outcomes. Sample size determination was primarily based on feasibility considerations and consistency with previously established experimental cerebral ischemia–reperfusion models, aiming to provide sufficient sensitivity to detect biologically meaningful intergroup differences.

All animals were maintained under controlled environmental conditions (23 ± 1 °C) with a 12 h light/dark cycle (lights on at 07:00) and had unrestricted access to standard laboratory chow and water. To minimize potential circadian influences, all experimental procedures were carried out between 08:00 and 12:00. Animals that developed infection during the study period or in whom adequate ischemia could not be reliably achieved were excluded prior to data acquisition and statistical analysis.

### 2.3. Anesthesia and Perioperative Monitoring

All animals received prophylactic cefazolin sodium (50 mg/kg, intraperitoneally; Sefazol, İstanbul, Turkey) 30 min prior to the induction of anesthesia. General anesthesia was induced via intraperitoneal (i.p.) administration of a combination of ketamine hydrochloride (60 mg/kg; Ketalar^®^, Pfizer, İstanbul, Turkey) and xylazine (5 mg/kg; Rompun^®^, Bayer, İstanbul, Turkey). During the surgical procedures, physiological parameters—including arterial oxygen saturation, heart rate, and rectal temperature—were continuously monitored to ensure hemodynamic stability. Core body temperature was maintained at 37 ± 0.5 °C using a thermostatically regulated heating pad.

### 2.4. Euthanasia and Tissue Collection

All animals were euthanized at 48 h following the induction of cerebral ischemia–reperfusion (I/R) injury. Euthanasia was performed under deep anesthesia to ensure a humane and pain-free death. Deep anesthesia was induced via intraperitoneal administration of a high-dose ketamine (100 mg/kg) and xylazine (10 mg/kg) combination. Adequate anesthetic depth was confirmed by the complete absence of corneal and pedal withdrawal reflexes. Following confirmation of deep anesthesia, euthanasia was completed by exsanguination via cardiac puncture. All procedures were conducted in strict accordance with the AVMA Guidelines for the Euthanasia of Animals, ensuring compliance with internationally accepted standards for ethical animal research.

### 2.5. Experimental Ischemia–Reperfusion Model

All surgical procedures were performed under sterile conditions. Animals under general anesthesia were placed in the prone position; the surgical site was shaved and disinfected with povidone-iodine solution. Following a midline cervical incision, the bilateral common carotid arteries were carefully dissected without damaging the surrounding soft tissues.

In the sham group, the carotid arteries were only exposed and no occlusion was applied. Ischemia–reperfusion injury was created in the IR group by temporarily occluding the bilateral common carotid arteries for 10 min using microvascular clamps and then removing the clamps to restore reperfusion. Care was taken to standardize the duration and severity of ischemia throughout the occlusion period in experimental group.

Following reperfusion, the surgical site was closed according to anatomical planes, and the animals were closely monitored during the postoperative period. This ischemia–reperfusion model is considered a reproducible and reliable method for experimentally inducing ischemic–reperfusion injury in the cerebral hemisphere cortex and subcortical regions [15]. To validate the successful establishment of the global ischemia model, neurological status was assessed at 2, 6, and 24 h post-reperfusion. The scoring criteria were as follows: 0, no deficit; 1, failure to fully extend the left forelimb; 2, circling to the left; 3, falling to the left; and 4, no spontaneous walking with a depressed level of consciousness. Only animals with a score of 1 or higher were considered successful models of ischemia.

### 2.6. Biochemical Analysis

Blood samples were collected from the tail vein at four predefined time points: baseline (0 min; following anesthesia induction and preoperative preparation, prior to the induction of ischemia) and at 6, 24, and 48 h after the onset of reperfusion. The samples were centrifuged at 3600 rpm for 10 min, and the separated serum was aliquoted and stored at −80 °C until analysis.

Serum endocan (endothelial cell-specific molecule-1, ESM-1) levels were quantified using a commercially available enzyme-linked immunosorbent assay (ELISA) kit (Boster Biological Technology, Pleasanton, CA, USA; Catalog No. EK0753), according to the manufacturer’s instructions. The assay is based on a sandwich ELISA principle using microplates pre-coated with antibodies specific for endocan.

Briefly, serum samples and standards were added to the wells and incubated under the recommended conditions. After washing to remove unbound substances, an enzyme-linked detection antibody was applied, followed by the addition of a substrate solution. The optical density was measured using a microplate reader, and endocan concentrations were calculated based on the standard calibration curve.

### 2.7. Histopathological Evaluation

Animals were euthanized at 48 h following the induction of cerebral ischemia–reperfusion (I/R) injury for histopathological assessment. Brains were rapidly removed and fixed in 10% neutral buffered formalin, followed by routine tissue processing and paraffin embedding using an automated tissue processor (TP1020, Leica Biosystems, Nussloch, Germany).

Paraffin blocks were sectioned coronally at a thickness of 4 μm using a rotary microtome SM2000R, Leica Biosystems, Nussloch, Germany). For each animal, six serial sections were obtained specifically from the hippocampal region, including the CA1 and CA3 subfields, to ensure a standardized and representative evaluation of ischemia-sensitive neuronal populations.

For histological examination, sections were deparaffinized in xylene, rehydrated through graded ethanol solutions, and stained with Mayer’s hematoxylin and eosin (H&E). After staining, the slides were dehydrated, cleared, and coverslipped using Entellan mounting medium. All histological images were acquired using a light microscope equipped with a digital imaging system BX53, Olympus Corporation, Tokyo, Japan.

The hippocampus was selected as the primary region of interest due to its high susceptibility to ischemia–reperfusion-induced oxidative stress, excitotoxicity, and apoptotic neuronal loss, making it a reliable indicator of secondary brain injury.

Neuronal degeneration was evaluated semi-quantitatively by two experienced neuropathologists who were blinded to the experimental groups. Assessment was based on established morphological criteria, including nuclear shrinkage, pyknosis, hyperchromasia, and cytoplasmic eosinophilia [16]. A four-point grading system was used as follows:

- Score 0: Normal histological tissue structure without visible pathological changes.
- Score 1: Minimal changes.
- Score 2: Moderate changes with focal areas of nuclear pyknosis and cytoplasmic vacuolation.
- Score 3: Significant pathological changes, namely pyknosis or vacuolation in more than half of the neurons and loss of the characteristic structure of neuronal layers.

To minimize observer bias, all evaluations were performed independently by two blinded observers. Inter-rater reliability for the ordinal neuronal degeneration scores was analyzed using weighted Cohen’s kappa, which demonstrated excellent agreement (κ = 0.85), thereby supporting the robustness and reproducibility of the histopathological analysis.

### 2.8. Statistical Analysis

All statistical analyses were performed using SPSS software (version 22.0; IBM Corp., Armonk, NY, USA). A *p*-value < 0.05 was considered statistically significant for all analyses. The normality of continuous variables, including serum endocan levels, was assessed at each time point using the Shapiro–Wilk test. Based on the distribution results, data were expressed as mean ± standard error of the mean (SEM).

To compare serum endocan levels between the sham and cerebral I/R groups at each predefined time point (0, 6, 24, and 48 h), independent samples *t*-tests were applied. Temporal changes in serum endocan levels within the I/R group across the four time points were analyzed using one-way repeated measures analysis of variance (ANOVA).

A group × time interaction was not separately modeled, as the primary study aim focused on between-group comparisons at individual time points and within-group temporal changes over time. When the assumption of sphericity was violated, as assessed by Mauchly’s test, the Greenhouse–Geisser correction was applied. Where appropriate, significant ANOVA findings were further explored using Bonferroni-adjusted post hoc comparisons, with baseline (0 h) values serving as the reference point.

Histopathological scores were treated as ordinal variables and are presented as medians with ranges. The relationship between serum endocan levels at 48 h and histopathological degeneration scores was evaluated using Spearman’s rank correlation analysis. For all correlation analyses, 95% confidence intervals (CIs) were calculated to quantify the precision of the estimates.

## 3. Results

### 3.1. Serum Endocan Concentrations

At the baseline time point (0 min), serum endocan levels did not differ significantly between the sham and ischemia–reperfusion (I/R) groups (*p* = 0.814). However, following induction of I/R injury, circulating endocan concentrations were markedly elevated in the I/R group compared to the sham group at 6 h (*p* < 0.005), 24 h (*p* < 0.001), and 48 h (*p* < 0.001), as summarized in Table 1.

**Figure 2:**
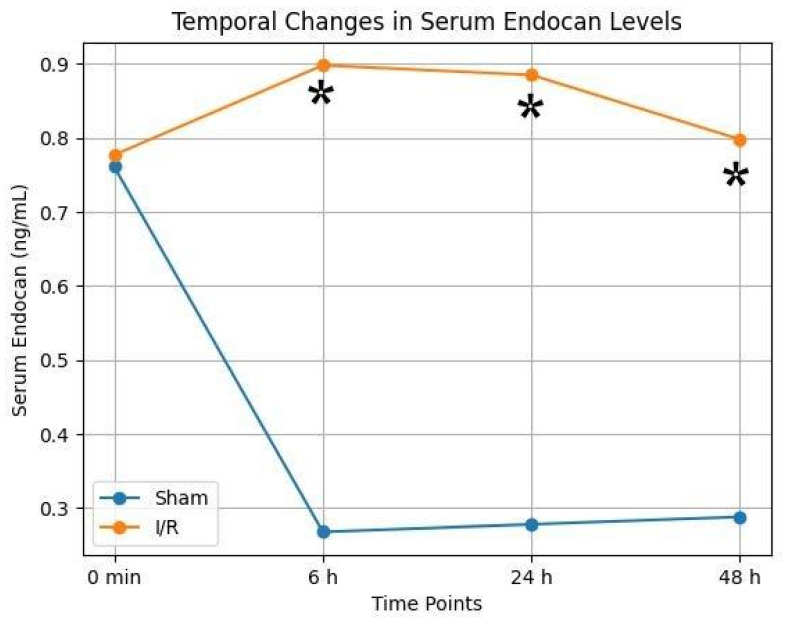
Temporal changes in serum endocan levels in the sham and I/R groups. Serum endocan concentrations (mean ± SEM) measured at baseline (0 min), 6, 24, and 48 h. The I/R group demonstrated a pronounced elevation in serum endocan levels at 6 h, followed by a gradual decrease at 24 and 48 h, though these levels remained significantly higher than at baseline and in the sham group. The sham group exhibited an initial reduction at 6 h, followed by minor fluctuations at subsequent time points. * *p* < 0.05 versus the sham group (I/R group at 6, 24, and 48 h).

In the intra-group analysis of the I/R cohort, serum endocan levels demonstrated a significant time-dependent increase. Relative to baseline values, endocan concentrations were significantly higher at 6 h (*p* = 0.003), 24 h (*p* < 0.001), and 48 h (*p* = 0.028) (Table 2). In contrast, no statistically significant fluctuations were observed in the sham group throughout the 48 h observation period. The temporal evolution of serum endocan levels in both groups is illustrated in Figure 2.

### 3.2. Association Between Neuronal Degeneration and Serum Endocan

**Figure 3:**
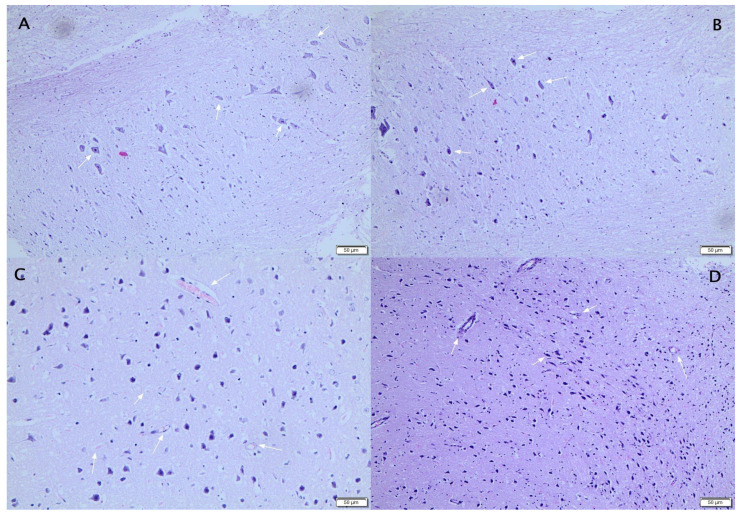
Histopathological alterations in the hippocampal CA1 region after experimental ischemia–reperfusion (I/R). (**A**) Representative section from a sham-operated rat (Grade 0) showing preserved cytoarchitecture of the CA1 region. Pyramidal neurons display normal morphology with round, euchromatic nuclei and clearly defined cytoplasm. (**B**) Section from an I/R group animal (Grade 1) demonstrating mild neuronal injury, characterized by scattered pyknotic neurons and subtle cytoplasmic changes. (**C**) Section from an I/R group animal (Grade 2) demonstrating moderate neuronal injury, including partial neuronal loss, increased numbers of pyknotic neurons (arrows), and cytoplasmic vacuolization (arrowheads), accompanied by early disruption of tissue organization. (**D**) Section from an I/R group animal (Grade 3) showing severe neurodegeneration in the CA1 region, with extensive neuronal loss; vacuolated neuropil; and the presence of shrunken, hyperchromatic, or ghost-like neurons (arrows). (Hematoxylin and eosin staining; original magnification ×200; scale bar = 50 µm).

Histopathological examination of hippocampal tissue was conducted to evaluate neuronal injury. The sham group exhibited no evidence of neuronal degeneration (median score = 0; Figure 3). In contrast, the I/R group showed varying degrees of neuronal damage, with degeneration scores ranging from 1 to 3 (median score = 2.5). These changes were characterized by features such as nuclear pyknosis and cytoplasmic vacuolization (Figure 3). Degeneration scores were significantly greater in the I/R group compared to the sham group (*p* < 0.001).

To further investigate the relationship between biochemical and histological findings, Spearman’s rank correlation analysis was performed between serum endocan levels and neuronal degeneration scores at 48 h within the I/R group. The results revealed a strong and statistically significant positive correlation (Spearman’s *ρ* = 0.857, [95% CI: 0.482–0.968]; *p* = 0.007; Table 3).

## 4. Discussion

The present study aimed to investigate the temporal profile of serum endocan levels following cerebral I/R injury and to evaluate its potential as a novel circulating biomarker for neurodegeneration. Our results demonstrate that serum endocan concentrations significantly rapidly elevate as early as 6 h post-reperfusion, followed by a gradual decline towards 24 and 48 h, though remaining significantly elevated above baseline throughout the 48 h observation period (Table 1 and Table 2). Most importantly, these elevated levels showed a strong positive correlation (*ρ* = 0.857, [95% CI: 0.482–0.968]) with the severity of hippocampal neuronal damage, suggesting that endocan is not only a marker of injury but also an indicator of its pathological extent.

Cerebral ischemia triggers a devastating cascade of biochemical failures, including bioenergetic collapse and disruption of ion homeostasis [17]. While rapid restoration of blood flow is the primary clinical goal, the subsequent reperfusion process paradoxically exacerbates tissue damage through oxidative stress and neuroinflammation [18]. This inflammatory milieu, characterized by the release of pro-inflammatory cytokines like TNF-α and IL-1β, is known to significantly upregulate endocan expression in activated vascular endothelial cells [19]. The marked elevation of serum endocan observed in our I/R group likely reflects this acute endothelial activation and the subsequent breakdown of the blood–brain barrier (BBB) associated with secondary injury.

Traditionally, neuroimaging techniques like CT and MRI are the gold standards for diagnosing stroke, yet they often face limitations regarding cost, infrastructure requirements, and a potential failure to detect early-stage pathologies [20]. While glial and neuronal markers such as NSE, S100B, and GFAP have been extensively studied, they primarily reflect cellular death. In contrast, endocan provides a unique perspective by directly reflecting endothelial dysfunction, which is a critical component of the I/R injury cascade [21]. Our findings align with previous research suggesting endocan as a marker for silent brain infarction and large-artery atherosclerotic stroke, while extending this knowledge to the specific context of acute I/R injury in a controlled rodent model. However, it is crucial to recognize that the 10 min BCCAO model primarily induces global cerebral ischemia, which may not fully recapitulate the complex pathophysiology of focal ischemic stroke in humans. Our decision for a 10 min occlusion was based on previous studies demonstrating that this duration reliably induces a reproducible, yet sub-lethal, ischemic injury in Sprague Dawley rats, allowing for the study of reperfusion injury without excessive mortality or irreversible damage that might obscure biomarker detection. This approach enabled us to focus on the early and dynamic changes in endocan in response to I/R injury. Therefore, direct clinical translation of these findings to focal ischemic stroke should be approached with caution, and future research should focus on validating these observations in models of focal ischemia.

The hippocampus was selected for histopathological analysis due to its extreme sensitivity to ischemia-induced oxidative stress and excitotoxicity [22]. The significant correlation between 48 h endocan levels and hippocampal degeneration scores—characterized by nuclear pyknosis and cytoplasmic vacuolization—highlights the biological relevance of this proteoglycan (Figure 3). Because endocan modulates leukocyte migration and the expression of adhesion molecules like ICAM-1, its rise may be actively involved in the inflammatory recruitment that leads to the observed neuronal death [23,24].

Despite the observed strong correlation between serum endocan levels and hippocampal neuronal damage, it is crucial to acknowledge a potential limitation regarding the specificity of endocan as a brain injury biomarker. Endocan, primarily secreted by activated vascular endothelial cells, is known to be expressed in various organs, including the lungs and kidneys [8]. Consequently, the elevated serum endocan levels detected in our cerebral I/R model could, at least in part, reflect a broader systemic inflammatory response rather than being exclusively indicative of brain-specific endothelial dysfunction or injury. While cerebral ischemia–reperfusion injury undoubtedly triggers local inflammation and blood–brain barrier disruption, systemic inflammation is also a well-documented consequence that can contribute to overall pathology [18]. Future studies should aim to differentiate the cerebral contribution to circulating endocan from potential extracerebral sources, perhaps through the concurrent measurement of other organ-specific endothelial activation markers or by investigating endocan expression directly within brain tissue versus peripheral organs following I/R injury. This distinction is vital for solidifying endocan’s utility as a specific diagnostic or prognostic tool for cerebral I/R injury.

Despite these promising results, this research was an exploratory pilot study with a relatively small sample size and a 48 h follow-up period. While we established a clear temporal increase, further studies are needed to determine the long-term prognostic value of endocan and its behavior over an extended recovery phase. We emphasize that these findings are preliminary and require validation in larger cohorts and more clinically relevant models of focal ischemia to confirm their translational potential.

Serum endocan serves as a sensitive, non-invasive, and biologically meaningful biomarker for cerebral I/R injury. Its rapid elevation and strong correlation with histopathological damage suggest that it could enhance the diagnostic and prognostic toolkit for managing acute ischemic stroke, potentially bridging the gap where traditional imaging may fall short.

## 5. Conclusions

This exploratory pilot study successfully demonstrated that serum endocan levels are significantly elevated in a rodent model of cerebral ischemia–reperfusion (I/R) injury, exhibiting a time-dependent increase as early as 6 h post-reperfusion and sustained elevation up to 48 h. Crucially, these elevated circulating endocan concentrations showed a strong positive correlation with the severity of hippocampal neuronal degeneration, as assessed by histopathological scoring. These findings suggest that serum endocan serves as a sensitive and biologically relevant circulating biomarker reflecting acute endothelial activation and subsequent neuronal damage in the context of cerebral I/R injury. The rapid and sustained elevation of endocan, coupled with its correlation to histopathological outcomes, positions it as a promising candidate for enhancing the diagnostic and prognostic toolkit for acute ischemic stroke, potentially offering a non-invasive method to monitor endothelial dysfunction and secondary injury processes where traditional neuroimaging may have limitations. However, we underscore the preliminary nature of these results, the limitations inherent in the global ischemia model, and the necessity for further validation in more complex models and larger sample sizes before clinical translation.

## 6. Limitations

Despite the promising findings, this study has several limitations. First, the sample size was small, which limits statistical power and generalizability, particularly for correlation analyses. The wide confidence interval for Spearman’s ρ, despite statistical significance, reflects the inherent instability of the point estimate with a small sample size. Larger cohorts are needed to validate the results. Second, the 10 min BCCAO model may not fully replicate the severity or complex pathophysiology of human ischemic stroke, and its adequacy for inducing consistent hippocampal damage should be further justified. Specifically, this global ischemia model differs from the focal nature of most acute ischemic strokes, limiting direct translational applicability to clinical focal stroke scenarios. Additionally, future studies should consider incorporating additional histological staining techniques, such as Nissl staining, to further corroborate the extent and specificity of neuronal damage, thereby enhancing the robustness of pathological evaluation. Furthermore, the absence of quantitative histopathological analyses, such as intact neuronal density measurements, may limit the objectivity of tissue injury assessment, although a blinded semi-quantitative grading system with high interobserver agreement was used to minimize subjectivity. Third, this study assessed only serum endocan, without including other biomarkers of inflammation, oxidative stress, or BBB integrity. The potential contribution of endocan from peripheral organs also remains unexplored. Fourth, the follow-up was limited to 48 h post-reperfusion, offering no insight into long-term endocan dynamics or functional outcomes. Finally, the cellular sources and regulatory mechanisms of endocan upregulation in cerebral I/R injury were not investigated, highlighting the need for mechanistic studies.

## Figures and Tables

**Table 1:** Between-group comparison of serum endocan levels (ng/mL) at different time points.

Time Point	Sham Group (Mean ± SEM)	I/R Group (Mean ± SEM)	Mean Difference (I/R/Sham) (95% CI)	*p*-Value
0 min (baseline)	0.761 ± 0.048	0.777 ± 0.047	0.016 (−0.118 to 0.150)	0.814
6 h	0.268 ± 0.037	0.898 ± 0.046	0.630 (0.514 to 0.746)	<0.005
24 h	0.278 ± 0.037	0.885 ± 0.037	0.607 (0.500 to 0.714)	<0.001
48 h	0.288 ± 0.037	0.798 ± 0.047	0.510 (0.388 to 0.632)	<0.001

Data are presented as mean ± standard error of the mean (SEM). Statistical comparisons between the sham and I/R groups at each time point were performed using an independent samples *t*-test. Statistically significant difference (*p* < 0.05) compared to the sham group at the corresponding time point.

**Table 2:** Within-group comparison of serum endocan levels (ng/mL) at different time points following I/R.

Time Point	Serum Endocan (ng/mL) in I/R Group (Mean ± SEM)	Mean Difference (vs. 0 min)	95% CI	*p*-Value
0 min (baseline)	0.777 ± 0.047	—	—	—
6 h	0.898 ± 0.046	0.121	(0.054 to 0.188)	0.003
24 h	0.885 ± 0.037	0.108	(0.048 to 0.168)	<0.001
48 h	0.798 ± 0.047	0.021	(0.003 to 0.039)	0.028

Data are presented as mean ± standard error of the mean (SEM). Statistical analysis was performed using a one-way repeated measures ANOVA, followed by post hoc pairwise comparisons against the baseline (0 min) value with Bonferroni correction. Statistically significant difference (*p* < 0.05) compared to the baseline measurement at 0 min. Overall ANOVA: F(1.58, 11.06) = 12.47, *p* = 0.001 (Greenhouse–Geisser-corrected) Bonferroni-corrected post hoc comparisons (α = 0.0167).

**Table 3:** Individual serum endocan levels (48 h) and neuronal degeneration scores in I/R group.

Animal ID	48 h Endocan (ng/mL)	Neuronal Degeneration Score
I/R-1	0.60	1
I/R-2	0.66	1
I/R-3	0.71	2
I/R-4	0.77	2
I/R-5	0.84	3
I/R-6	0.89	3
I/R-7	0.93	3
I/R-8	0.98	3

Spearman’s correlation: *ρ* = 0.857 (95% CI: 0.482–0.968), *p* = 0.007.

## Data Availability

The datasets generated and analyzed during the current study are available from the corresponding author upon reasonable request.

## Abbreviations

ANOVA	Analysis of Variance
ARRIVE	Animal Research: Reporting of In Vivo Experiments
AVMA	American Veterinary Medical Association
BBB	Blood–Brain Barrier
BCCAO	Bilateral Common Carotid Artery Occlusion
CA1	Cornu Ammonis 1
CA3	Cornu Ammonis 3
CI	Confidence Interval
CT	Computed Tomography
ELISA	Enzyme-Linked Immunosorbent Assay
ESM-1	Endothelial Cell-Specific Molecule-1
GFAP	Glial Fibrillary Acidic Protein
H&E	Hematoxylin and Eosin
ICAM-1	Intercellular Adhesion Molecule-1
IL-1β	Interleukin-1 Beta
I/R	Ischemia–Reperfusion
MRI	Magnetic Resonance Imaging
NSE	Neuron-Specific Enolase
PET	Positron Emission Tomography
S100B	S100 Calcium-Binding Protein B
SEM	Standard Error of the Mean
SPSS	Statistical Package for the Social Sciences
TNF-α	Tumor Necrosis Factor-Alpha
VCAM-1	Vascular Cell Adhesion Molecule-1
VEGF	Vascular Endothelial Growth Factor
